# Retrospective validation study of a machine learning-based software for empirical and organism-targeted antibiotic therapy selection

**DOI:** 10.1128/aac.00777-24

**Published:** 2024-08-28

**Authors:** Maria Isabel Tejeda, Javier Fernández, Pablo Valledor, Cristina Almirall, José Barberán, Santiago Romero-Brufau

**Affiliations:** 1 Infectious Diseases Unit, Hospital Universitario HM Montepríncipe, Madrid, Spain; 2 Research and Innovation Department, Pragmatech AI Solutions, Oviedo, Spain; 3 Microbiology Department, Hospital Universitario Central de Asturias, Oviedo, Spain; 4 Microbiology and Infectious Pathology, ISPA, Oviedo, Spain; 5 Functional Biology Department, Universidad de Oviedo, Oviedo, Spain; 6 Department of Laboratory Medicine, HM Hospitales, Madrid, Spain; 7 HM Faculty of Health Sciences, University Camilo Jose Cela, Madrid, Spain; 8 Department of Otorhinolaryngology–Head & Neck Surgery, Mayo Clinic, Rochester, Minnesota, USA; 9 Department of Biostatistics, Harvard T.H. Chan School of Public Health, Harvard University, Boston, Massachusetts, USA; Johns Hopkins University School of Medicine, Baltimore, Maryland, USA

**Keywords:** machine learning, antibiotic therapy, antimicrobial stewardship

## Abstract

Errors in antibiotic prescriptions are frequent, often resulting from the inadequate coverage of the infection-causative microorganism. The efficacy of iAST, a machine-learning-based software offering empirical and organism-targeted antibiotic recommendations, was assessed. The study was conducted in a 12-hospital Spanish institution. After model fine-tuning with 27,531 historical antibiograms, 325 consecutive patients with acute infections were selected for retrospective validation. The primary endpoint was comparing each of the top three of iAST’s antibiotic recommendations’ success rates (confirmed by antibiogram results) with the antibiotic prescribed by the physicians. Secondary endpoints included examining the same hypothesis within specific study population subgroups and assessing antibiotic stewardship by comparing the percentage of antibiotics recommended that belonged to different World Health Organization AWaRe groups within each arm of the study. All of iAST first three recommendations were non-inferior to doctor prescription in the primary endpoint analysis population as well as the secondary endpoint. The overall success rate of doctors’ empirical treatment was 68.93%, while that of the first three iAST options was 91.06% (*P* < 0.001), 90.63% (*P* < 0.001), and 91.06% (*P* < 001), respectively. For organism-targeted therapy, the doctor’s overall success rate was 84.16%, and that of the first three ranked iAST options was 97.83% (*P* < 0.001), 94.09% (*P* < 0.001), and 91.30% (*P* < 0.001), respectively. In empirical therapy, compared to physician prescriptions, iAST demonstrated a greater propensity to recommend access antibiotics, fewer watch antibiotics, and higher reserve antibiotics. In organism-targeted therapy, iAST advised a higher utilization of access antibiotics. The present study demonstrates iAST accuracy in predicting antibiotic susceptibility, showcasing its potential to promote effective antibiotic stewardship.

**CLINICAL TRIALS:**

This study is registered with ClinicalTrials.gov as NCT06174519.

## INTRODUCTION

Bacterial infections are one of the main causes of mortality worldwide both in low- and high-income countries. They cause more than 6 million deaths per year and ranked as the second leading cause of death globally in 2019 (1).

The emergence and spread of antimicrobial bacterial resistance (AMR) represent one of the most important public health challenges worldwide (2). In recent years, many international organizations have recognized the importance of taking action to prevent the spread of antibiotic-resistant pathogens (3). In 2015, the World Health Organization (WHO) designed a global plan to face this problem, with five objectives including optimizing the use of antimicrobials (4). Antibiotic stewardship programs have become an essential tool to optimize antimicrobial treatment and reduce bacterial resistance to antibiotics worldwide (5). Stewardship policies seek to achieve a delicate balance between prioritizing antibiotics with minimal ecological impact and ensuring sufficient coverage of the causative agent of infection. The latter arises from well-established knowledge that delayed coverage of the infecting agent can lead to complications and increased mortality rates in patients (6–8). Despite efforts to address this issue and the enactment of stewardship policies, errors persist in antibiotic prescriptions, often stemming from the inadequate coverage of the causative microorganism (9–11).

In the course of a suspected infection, antibiotics are initially prescribed empirically; several hours later, the treatment can be refined after knowing the causative agent (organism-targeted therapy); however, it typically takes 24–48 h to know the results of the antibiotic susceptibility testing (AST) result, which will provide the resistance pattern (12). In recent years, clinical microbiology laboratories have integrated new diagnostic techniques enabling the rapid identification of infection-causing agents. However, this advancement often results in a time gap between the identification of the causal agent and the determination of its antibiotic susceptibility (13). The prompt identification of the etiological agent presents an opportunity to implement timely organism-targeted antimicrobial therapy, thereby reducing the empirical nature of the initial treatment for bacterial infections. This approach is further enhanced by leveraging cumulative antibiogram data (14). Artificial intelligence holds great opportunity as a valuable tool in this objective; in fact, several studies have assessed the effectiveness of employing machine learning (ML) techniques on aggregated antibiogram data and clinical records to guide empirical antibiotic therapy, yielding promising results (15–19). The majority of these studies primarily assess software developed as an in-house tool for specific institutions and are concentrating solely on a specific type of infection, as well as a limited number of antibiotics and microorganisms (17, 19). On the other hand, many of them have been validated from a mathematical perspective, but not through a clinical investigation carried out by independent researchers.

The aim of the present study was to evaluate iAST (Pragmatech AI Solutions, Oviedo, Spain), a ML-based software that provides empirical and organism-targeted antibiotic recommendations, and to compare its accuracy with doctors’ therapy using AST results as the gold standard.

## MATERIALS AND METHODS

### Setting

The study was carried out in the HM hospital system, a private hospital system in Madrid, Spain, comprised of secondary and tertiary care hospitals, with 1,785 total beds across 12 hospitals.

### iAST software

The iAST software (Pragmatech AI Solutions) is an AI-based algorithm that provides antibiotic recommendations for the empirical treatment of urinary tract infection, bacteremia, and nosocomial pneumonia. It can also provide organism-targeted therapy recommendations for any infection type. The software was developed as a single machine learning model that encompasses a broad spectrum of infectious syndromes. This global model takes into account the full range of relevant microorganisms and antibiotics. The AI model embedded is based on a classification approach implemented using boosting decision trees as a ML technique supplemented with a random bagging strategy and was initially developed by using data from scientific literature and public databases. The model considers as features a set of patient-related inputs (patient age, gender, institution, hospital ward or section where the patient is being treated, and microorganism in case of organism-targeted therapy), and as output, the iAST ML model generates a probability-based ranking of recommended antibiotics specific to the patient (please see Fig. S1 for an illustrative example). The architecture of the AI model is based on two processes: an offline batch process where the AI model is adjusted to the data, generating a static model artifact, and an online process where the inference of the AI model is performed over specific patient data. The adjustment of the model parameters based on the local data was performed considering the 12 hospitals of the entire healthcare institution at once. During the study, the software was run as standalone software, and input data for the few data elements required for each patient were entered manually.

The software algorithm also incorporates data on antibiotic resistance from scientific literature and public databases, data scrapped from publications in PubMed containing information on antibiotic resistance in different areas, and European Committee on Antimicrobial Susceptibility Testing (EUCAST) documents including intrinsic resistances of microorganisms, as well as local susceptibility data specifically derived from historical antibiogram results from the client healthcare institution extracted from the laboratory information system (LIS), which includes the aforementioned patient information. Once the data have been extracted and preprocessed, it is automatically integrated into the algorithm.

As an additional insight, iAST incorporates recommended antibiotic doses aligned with those established by EUCAST guidelines (www.eucast.org).

### Model fine-tuning and performance measurement

**Fig 1:**
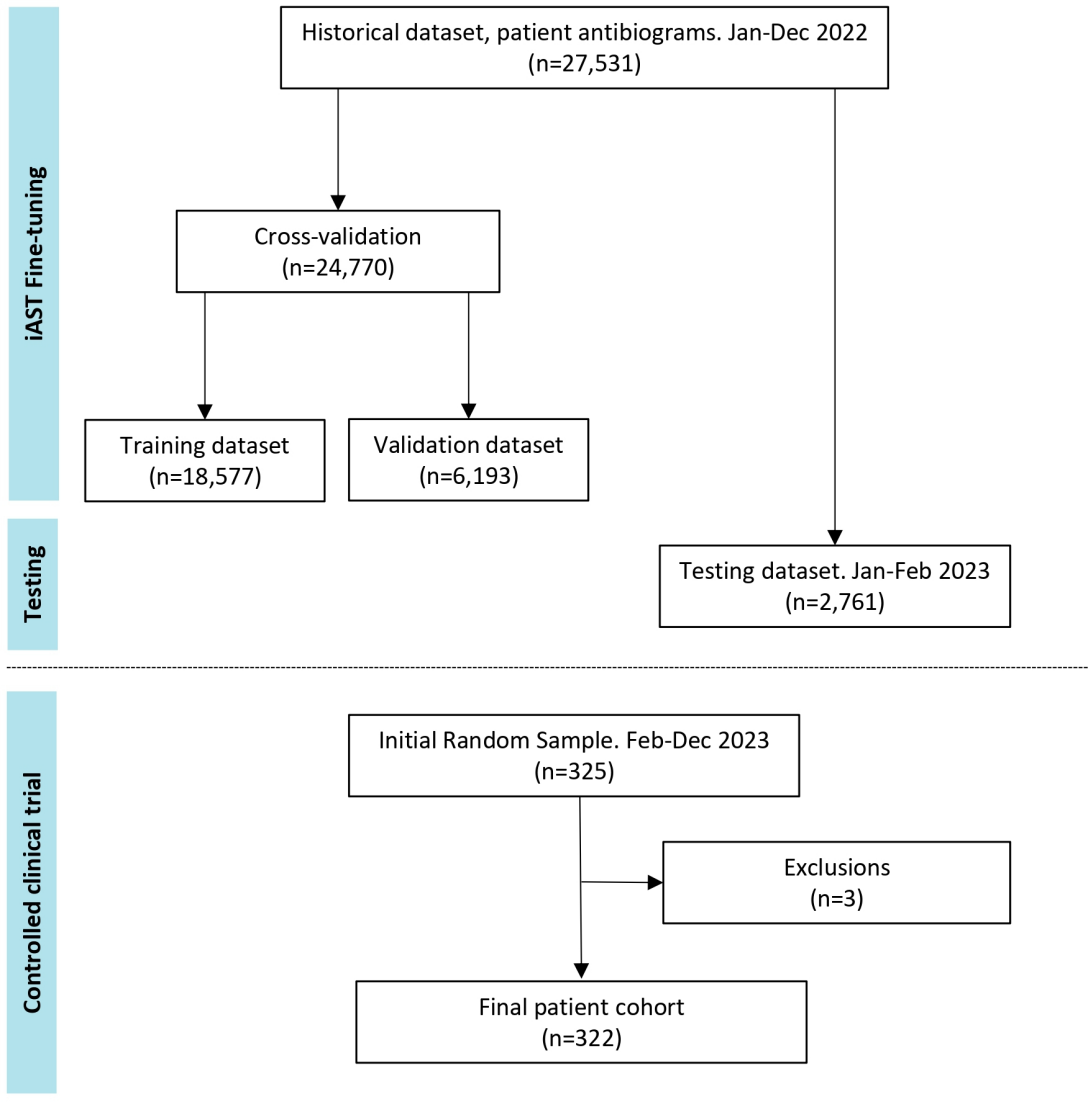
EVIAST data flow diagram. The diagram shows the different patient cohorts and data sets. The EVIAST study consisted of two phases: the first phase was the fine-tuning of the machine learning model to the local institution’s data. The second phase was the clinical evaluation of the iAST model and was carried out by independent investigators. Three patients were excluded due to screening failures; specifically, two cases did not receive any antibiotic therapy, and in a single case, the participant was treated before the study initiation date.

The model was fine-tuned with antibiogram data from patients who had a hospital admission from 1 January 2022 to 31 December 2022, with any type of infection. The cumulative antibiograms of the 20 most frequent bacteria recovered from patients admitted to HM hospitals during that period are shown in Tables S1 (Gram-negative bacteria) and S2 (Gram-positive bacteria). These data were extracted automatically from the LIS and included the microorganisms and their AST results along with the demographics (age and sex) of the patient, type of sample, type of infection, and admission location, including the hospital and hospital ward. The data set was split into the training, validation, and testing data sets: for the training data set, a sample of 75% of the historical data in 2022 was selected to build the model by a cross-validation procedure; the remaining 25% sample of historical data in 2022 was used as the validation data set to detect potential overfitting during the training stage process. Data from patients treated from 1 January 2023 to 14 February 2023 were used as a testing set to provide quantitative measurements of the behavior of the model with recent patients (Fig. 1). Performance metrics, including accuracy, precision, recall, area under the curve (AUC), and log loss (quantifying the disparity between predicted probabilities and actual labels), were calculated on both training and testing data sets to assess the efficacy of the algorithm.

### Study design and participants

The retrospective validation study (EVIAST) was carried out from August to December 2023 in the HM Hospitals, subsequent to the model being fine-tuned with local data. Patient cases from February 2023 onwards were selected. The identification of cases was accomplished through a review of hospital admission reasons and the database of positive culture results. Subsequently, it was verified that the patients exhibited clinical signs and symptoms consistent with infection. The study enrolled adult patients who met one of the following inclusion criteria: (i) presented with a urinary tract infection (UTI), (ii) experienced a blood stream infection (BSI), (iii) were admitted to the hospital intensive care unit (ICU) and presented with ventilator-associated tracheobronchitis (VAT) or pneumonia, or (iv) encountered another type of infection and had a bacterium identified. The study excluded patients who met any of the following criteria: (i) concomitant infections, defined as more than one infection source at the same time, (ii) subjects experiencing infections with no bacterial etiology (fungal or viral infections), (iii) subjects with infections lacking microbiological documentation (i.e., AST results), and (iv) subjects under antibiotic combination therapy. Among all patients meeting those criteria, a sample of 325 consecutive cases was selected by independent medical researchers. Specifically, they selected the first cases of each pathology within the study period from these hospitals, ensuring a minimum representation of 69 subjects from each infection subgroup. In the selected sample, a thorough review of patients’ medical records was performed to extract the patient demographics, type and date of infection, antibiotic prescribed by the attending physician, and microbiological results including AST results.

The trial’s design was formulated by representatives from the sponsor (Pragmatech AI Solutions, Oviedo, Spain) and subjected to review and monitoring by an independent Contract Research Organization (NAMSA, Northwood, OH, USA). Data were recorded in an electronic case report form (Xolomon Tree, Madrid, Spain) by investigators and site personnel.

### Patient selection and measures

For eligible subjects, independent investigators from HM Hospitales utilized the iAST tool installed in the institution to predict recommended antibiotics for both empirical and organism-targeted therapy. The first three antibiotics from the ranking that had been tested in the HM group’s AST results were documented. Additionally, the predicted coverage probability for each antibiotic was duly recorded. Investigators then cross-referenced the final microbiological reports, noting whether the recovered bacteria were susceptible to the prescribed drugs by doctors and predicted by iAST, based on the final AST results.

Finally, for the antibiotic prescribed by the clinicians, both as empirical therapy and as organism-targeted therapy, the investigators registered the predicted probability of susceptibility according to iAST, as well as the AST results for those antibiotics.

### Endpoints and statistical analysis

The primary goal of the clinical investigation was to demonstrate the non-inferiority of iAST compared to physicians for the prescription of both empirical and organism-targeted antibiotic therapy in patients with common infectious diseases. The primary endpoint was the overall success rate (as confirmed by the AST results) of iAST’s first, second, and third antibiotic recommendations, compared with the physician recommendations. Additionally, as a secondary endpoint, the identical hypothesis was examined within the four distinct study population subgroups, namely, UTI, BSI, pneumonia/VAT, and other infections. An additional predefined secondary endpoint was the utilization rates of antibiotics by the WHO AWaRe classification for iAST recommendations compared with physician predictions. The WHO AWaRe classification takes into account the impact of different antibiotics on antimicrobial resistance categorizing them into the access, watch, and reserve categories (20).

The study was designed to ensure adequate power (a minimum of 80%) to test the primary and secondary hypotheses against a non-inferiority margin of 5%, while maintaining a confidence level of 95%. The rate of success of the physician prescription (both for the empirical and organism-targeted therapy) was calculated taking the AST results as the gold standard. The iAST success rate was calculated by independently comparing each of the three top-ranked recommended antibiotics with the AST results: if the antibiogram determined that the causative organism was resistant to any of the three recommended antibiotics, this was considered a failure in the recommendation. In cases where clinicians did not switch the empirically prescribed drug to an alternative for organism-targeted therapy, the drug recommended by iAST for organism-targeted therapy was compared with the clinician’s empirical treatment.

Two-sided 95% confidence intervals (CIs) for the difference between treatments were calculated using the unstratified method of Miettinen and Nurminen. The non-inferiority endpoint (for both primary and secondary endpoints) was defined as whether the lower limit of the two-sided 95% CI for the treatment difference was greater than 5%. Additionally, a *P*-value was computed for the corresponding one-sided non-inferiority hypothesis test.

Statistical comparisons for each AWaRe level were performed between the doctors and the first-, second-, and third-ranking recommendations by iAST, by using the Chi-squared test.

The mean probabilities predicted by iAST for the antibiotic prescribed by the doctors were compared using the Welch two-sample *t*-test between cases where the bacterium exhibited susceptibility and cases where it showed resistance, according to the AST results.

All statistical analyses were performed by using RStudio 2023.06.0+421, R version 4.3.1, and SAS Statistical Software (version 9.4).

## RESULTS

### ML model evaluation metrics

**Fig 2:**
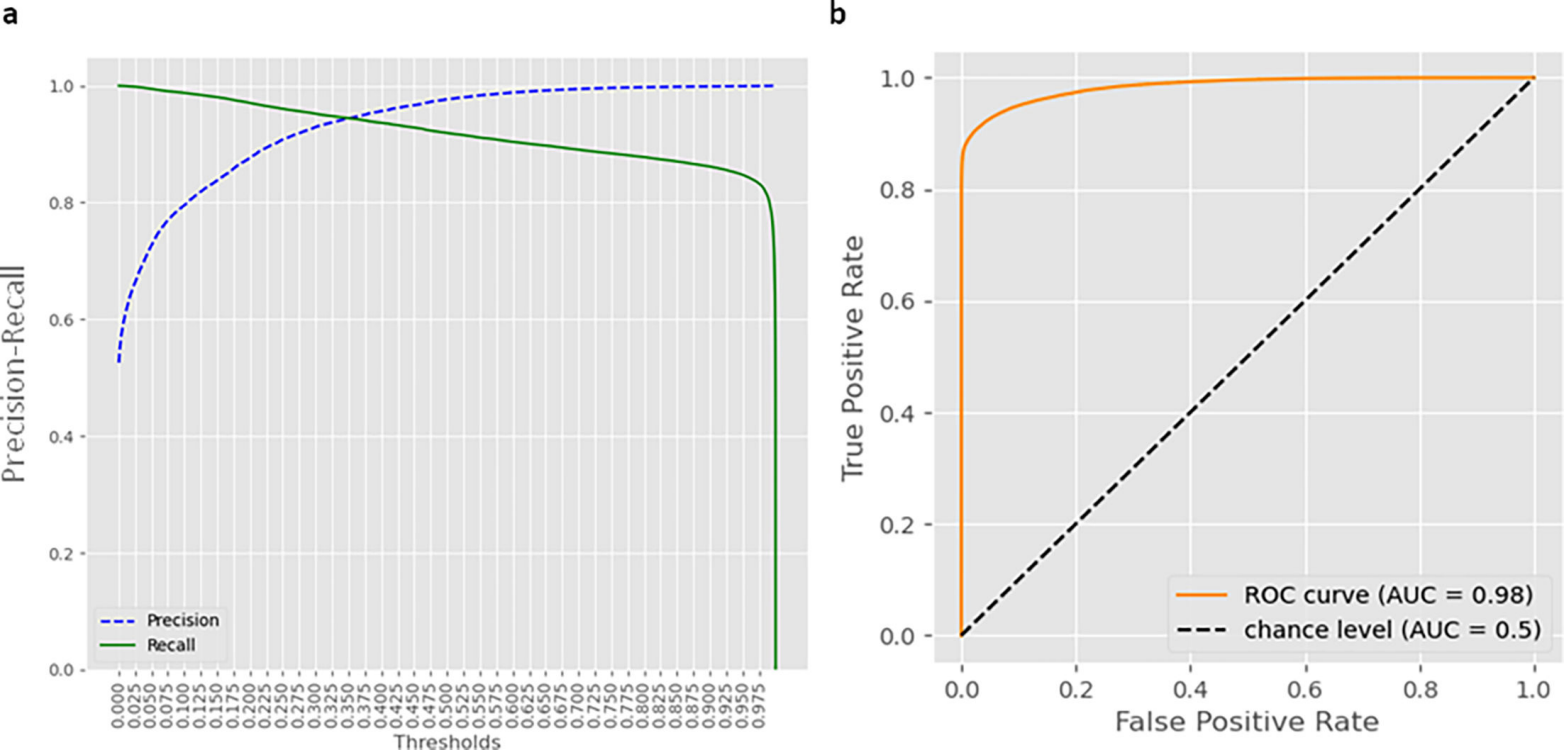
Machine learning model evaluation after fine-tuning. (**a**) Precision and recall metrics for the identification of resistant microorganisms depending on the discrimination threshold after fine-tuning with local data. (**b**) ROC curve for iAST top antibiotic recommendation, on the testing data set.

Performance metrics in the validation and testing data sets were similar: in the validation data set, iAST achieved a mean accuracy of 88%, a precision of 97% when identifying antibiotic susceptible bacteria, a recall (sensitivity) of 97% for resistant bacteria, and a log loss of 0.143, whereas in the testing data set, iAST achieved an accuracy of 89%, 0.148 for log loss, and the same values for the remaining metrics. Regarding the receiver operating characteristics (ROC) curve, the model achieved an AUC of 0.98, showing great discrimination between susceptible and resistant microorganisms. Precision/recall and AUC ROC curves, in the validation data set, are shown in Fig. 2.

### Cohort description

Out of the 325 adult participants assigned to the study after model fine-tuning, three were excluded due to screening failures (Fig. 1). In the overall population, the mean age was 68.87 years with males comprising 57.76% of the participants. They received care at one of eight different hospitals within the HM group, with 120 (37.27%) being outpatients in the emergency department and 202 (62.73%) being hospitalized patients admitted to one of eight different wards, largely in internal medicine/infectious diseases (129, 63.86%). Among the episodes of care included, 93 (28.88%) were cases of BSIs, 73 (22.67%) were UTIs, 69 (21.43%) were pneumonia/VAT, and 87 (27.02%) were classified as other infections (Table 1). All the selected infections were monomicrobial and were attributed to as many as 42 different microorganisms (Fig. S2). The rates of antibiotic resistance in these bacteria were similar to the general resistance rates shown in Tables S1 and S2. Regarding the presence of multidrug-resistant microorganisms in the cohort: 21.9% (9/41) of *Staphylococcus aureus* were resistant to methicillin; 14.8% (4/27) and 66.7% (2/3) of *Pseudomonas aeruginosa* and *Acinetobacter baumannii* isolates, respectively, were resistant to carbapenems; and 19% (29/150) and 2% (3/150) of enterobacterales isolates produced extended-spectrum beta-lactamases and carbapenemases, respectively.

**TABLE 1:** Main features of patients and infections included in the study

Characteristic	Value
Age (mean)	68.87
Gender	
Female	136 (42.24)
Male	186 (57.76)
Hospital attention	
Emergency department (outpatients)	120 (37.27)
Hospitalization	202 (62.73)
Hospitalization ward	
Anesthesia/postoperative intensive care unit	1 (0.49)
Gastroenterology/general surgery	1 (0.49)
General intensive care unit	65 (32.18)
Hematology	1 (0.49)
Internal medicine/infectious diseases	129 (63.86)
Neurology/neurosurgery	2 (0.99)
Oncology	2 (0.99)
Orthopedics/traumatology	1 (0.49)
Type of infection	
Bacteremia/sepsis	93 (28.88)
Urinary tract infection	73 (22.67)
Complicated^*a*^	57 (78.08)
Uncomplicated^*a*^	16 (21.91)
Nosocomial lower respiratory infection	69 (21.43)
Pneumonia	52 (75.36)
Ventilator-associated pneumonia	38 (73.07)
No ventilator-associated pneumonia	14 (26.92)
Tracheobronchitis	17 (24.64)
Others^*b*^	87 (27.02)
Bone and joint infection	6 (6.89)
Gastrointestinal infection	7 (8.05)
Lower respiratory system infection^*c*^	2 (2.29)
Reproductive tract infection	2 (2.29)
Skin and soft tissue infection	42 (48.28)
Surgical site infection	28 (32.18)

^*a*^ An uncomplicated urinary tract infection was considered in young women without any functional or anatomical anomalies in the urinary tract, whereas complicated infections were considered as those occurring in individuals with such anomalies or in other demographic groups.

^*b*^ CDC/NHSN definitions were used to classify the infections (https://www.cdc.gov/nhsn/pdfs/pscmanual/17pscnosinfdef_current.pdf).

^*c*^ Community-acquired lower respiratory system infections.

### Clinical study outcomes

**Fig 3:**
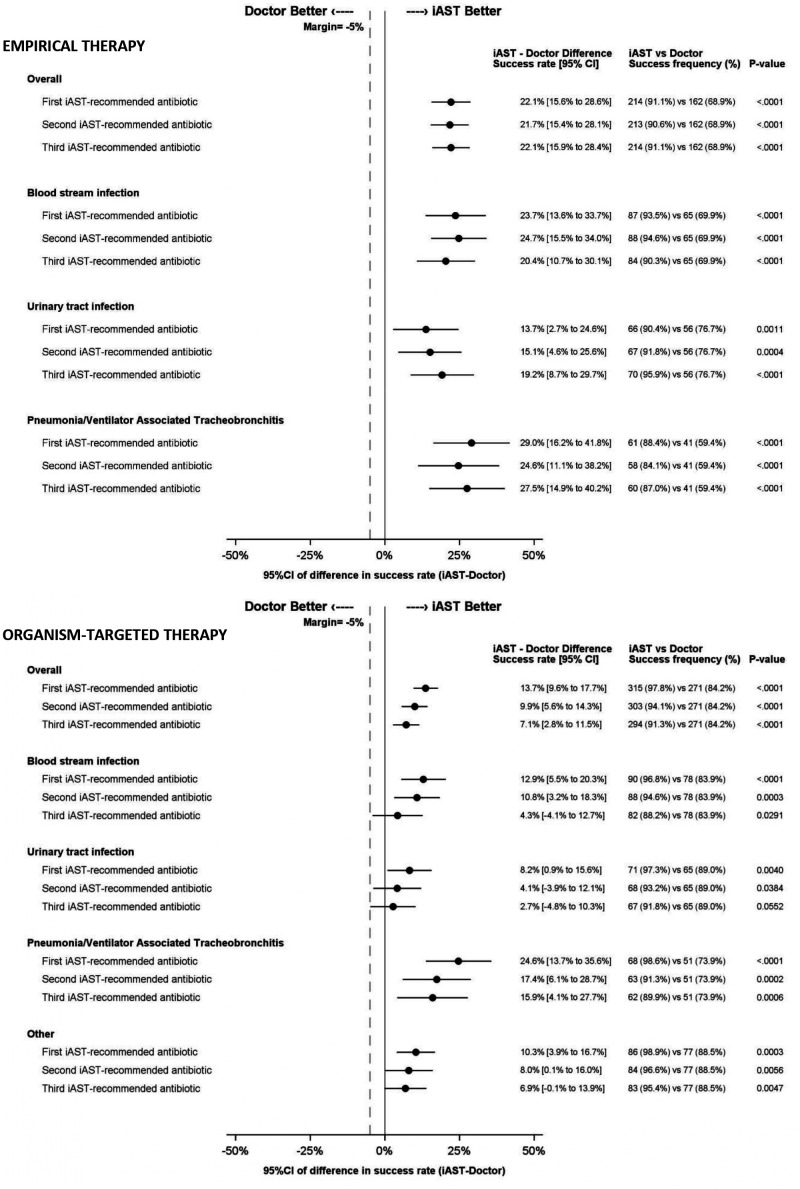
Study outcomes for empirical and organism-targeted therapy. Success is defined as a case in which the antibiotic prescription or recommendation was effective against the causative agent according to antibiotic susceptibility testing results.

iAST first three recommendations were non-inferior to doctor prescription in the primary endpoint analysis population as well as in the secondary endpoint including infection subgroup analysis (Fig. 3). The overall success rate of doctors’ empirical treatment overall was 68.93%, while that of the first three iAST options was 91.06% (*P* < 0.001), 90.63% (*P* < 0.001), and 91.06% (*P* < 0.001), respectively. For organism-targeted therapy, the doctor’s overall success rate was 84.16%, and that of the first three ranked iAST options was 97.83% (*P* < 0.001), 94.09% (*P* < 0.001), and 91.30% (*P* < 0.001), respectively. The success rates of doctors and iAST for each type of treatment and infection are showed in Fig. 3. All the drugs recommended by iAST were included in the hospital drug formulary. Failures on empirical and organism-targeted therapy committed by doctors and first three ranked iAST recommendations are detailed in Tables S3 and S4, respectively. When the doctor accurately prescribed empirical treatment, the iAST-predicted mean of predicted probabilities of susceptibility for the prescribed antibiotic was 85.23%. In contrast, in the cases when the empirical treatment failed, the mean was 75.99% (*P* < 0.001). In the context of organism-targeted treatment, the software predicted average coverage percentages of 88.94% for successful cases and 61.07% for failures (*P* < 0.001).

**Fig 4:**
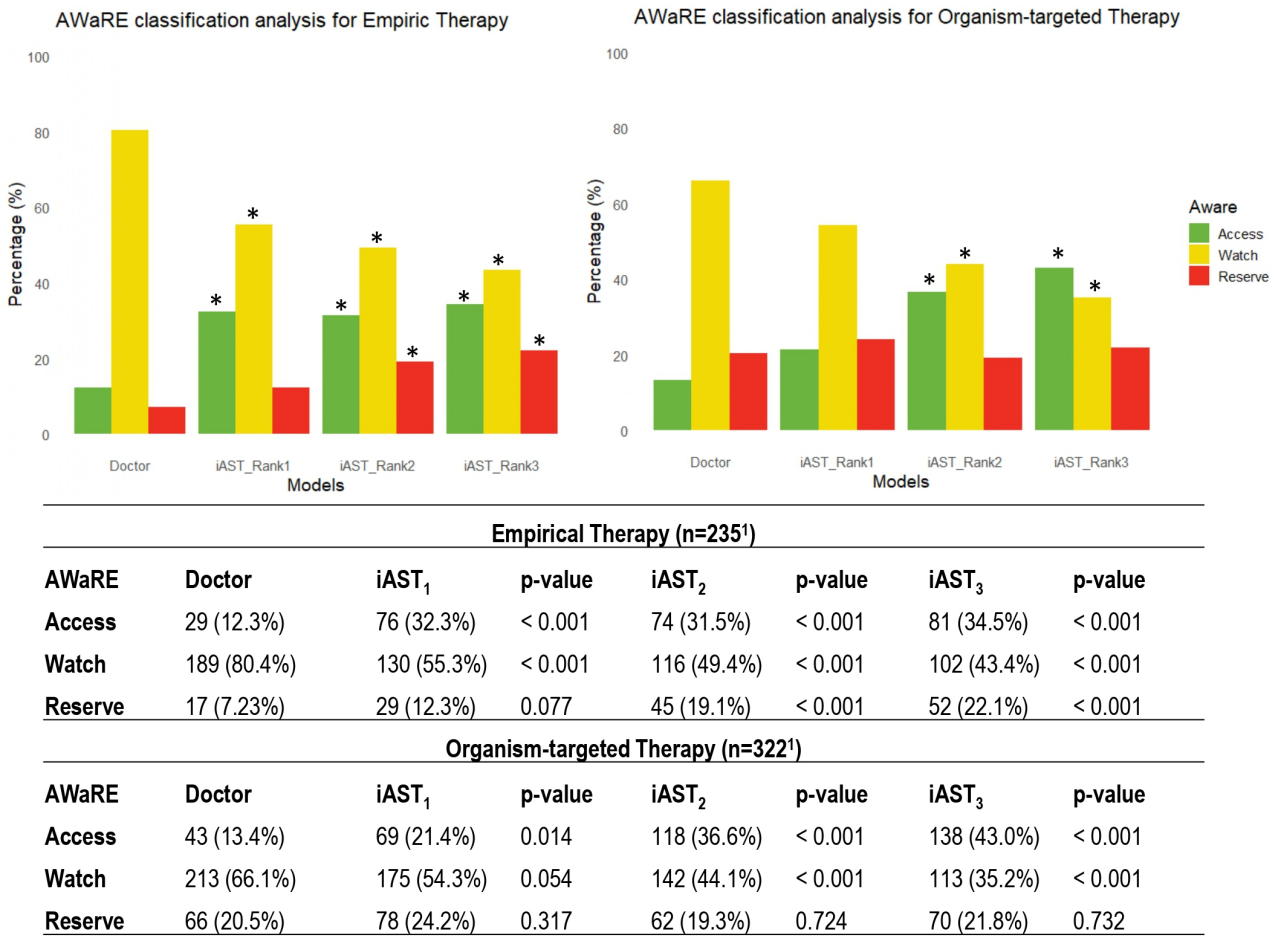
WHO AWaRe group analysis. The graph illustrates percentages of prescribed/recommended antibiotics by their WHO AWaRe group for antibiotic stewardship. The access group is comprised of antibiotics considered the first line from a stewardship perspective, while the reserve group is recommended as a last resort for patients with infections likely resistant to antibiotics in the other two groups. iAST_1_, iAST_2_, and iAST_3_ are the antibiotics recommended by iAST as first, second, and third choices, respectively. An asterisk denotes a statistically significant difference when compared to the same group for the physician prescription. ^1^The number of antibiotics in the organism-targeted group is higher than in the empirical group. This difference arises from the inclusion of patients with infections beyond urinary tract infection, blood stream infection, and pneumonia/ventilator-associated tracheobronchitis in the former.

In the context of employing/recommending antibiotics based on the AWaRe WHO classification for empirical therapy, iAST exhibited an increase in recommending both reserve and access antibiotics compared to those prescribed by physicians. However, this trend was accompanied by a decrease in the use of antibiotics in the watch group (Fig. 4). Conversely, in organism-targeted therapy, no significant differences were observed in the utilization/recommendation of reserve antibiotics between doctors and iAST; nonetheless, iAST recommended a higher number of antibiotics from the access group in comparison to medical practitioners (Fig. 4). Reserve category antibiotic recommendations were predominantly observed in Gram-positive bacterial infections, owing to the common suggestion of antibiotics such as linezolid or daptomycin, particularly for coagulase-negative staphylococci and enterococci (notably *Enterococcus faecium*). Another case of reserve category antibiotic recommendation involved infections in ICU patients with conditions such as BSI or pneumonia, particularly those caused by *Pseudomonas aeruginosa* in which ceftolozane/tazobactam was frequently recommended. Due to the baseline resistances in this species, the software placed this antibiotic at the top of the ranking. The frequency of antibiotics from the AWaRe reserve category prescribed by the doctor or recommended by the iAST software, by type of molecule and infection, is detailed in Table S5.

## DISCUSSION

EVIAST demonstrated the non-inferiority of ML software when compared to traditional doctor prescriptions, showcasing the effectiveness of an artificial intelligence-based tool in guiding antibiotic therapy for a broad spectrum of infections. The findings showcased a significant accuracy of iAST predictions, ensuring the appropriateness of antibiotic treatments for common infection-causing agents, as compared to decisions made by physicians. This holds significant importance, particularly given that a substantial number of patients underwent treatment in infectious diseases hospital wards.

Several prior studies have underscored the promising potential of ML in precisely predicting AMR. However, these studies were validated for specific infections, microorganisms, and/or antibiotics and mostly within the realm of research and from a mathematical perspective. Yelin et al. leveraged data sets of UTIs and established robust associations between AMR and patient demographics, past urine cultures, and drug purchase history (16). A mathematically retrospective application of these algorithms demonstrated a significant reduction in the risk of mismatched treatment compared to the current standard of care. Nevertheless, this algorithm was specifically employed for a single infection (UTIs) and a restricted set of six antibiotics prescribed for these infections, lacking any comparison with physician prescriptions within a clinical investigation (16, 21). In the same way, Moran et al. assessed the performance of XGBoost’s model in predicting antibiotic susceptibility of *Escherichia coli*, *Klebsiella pneumoniae*, and *Pseudomonas aeruginosa* recovered from UTI and BSI against amoxicillin/clavulanic and piperacillin/tazobactam. However, their results yielded limited metrics, with an AUC of 0.70 for the ML model (22). Similarly, in the context of BSI, Elligsen et al. crafted a logistic regression model to anticipate the likelihood of susceptibility among Gram-negative bacteria to five antibiotics (23). Several other noteworthy studies have employed ML techniques to assess the likelihood of isolates from specific infections harboring resistance mechanisms, such as extended-spectrum beta-lactamases (24, 25), or being multidrug-resistant (26). In a recent systematic review comparing performance metrics across various models aimed at antibiotic resistance prediction, it was noted that the majority achieved AUC ROC values ranging from 0.6 to 0.9 (17), with a single study reporting metrics with an AUC >0.9 (0.93). This particular study focused on predicting AMR in three microorganisms—*Acinetobacter baumannii, K. pneumoniae*, and *P. aeruginosa* (27).

The iAST system demonstrated AUC values of 0.98 in the present evaluation, used on a large number of microorganisms and antibiotics. The superiority observed in comparison to previous works evaluating ML models for predicting AMR could be attributed to the software’s reliance on a hybrid model. iAST integrates ML with clinical expertise, incorporating information on intrinsic resistances, resistances documented in the literature, clinical guidelines, and other relevant factors. The comprehensive nature of this approach likely played a significant role in achieving so outstanding metrics.

With respect to the management of infections within the institution, physicians adhere to the European and Spanish infectious disease society guidelines, tailoring antibiotic selection to the local microbial ecology. Doctors’ error rates, as documented in several studies (9–11), were found to be consistently high, particularly among patients with pneumonia and VAT admitted to the ICU. This rate may be attributed to the presence of multidrug-resistant isolates, including cases involving methicillin-resistant *S. aureus* and multidrug-resistant *Enterobacterales*, *Pseudomonas aeruginosa*, and *Stenotrophomonas maltophilia*. A recent comprehensive study conducted across 333 ICUs in 52 countries revealed that in cases of sepsis, antimicrobial therapy was deemed adequate within the first 24 h in only 51.5% of instances (8). The researchers attributed this low rate of coverage of the etiological agent to AMR, especially in infections caused by the aforementioned microorganisms.

Interestingly, the increased success rates with iAST over physicians did not involve the indiscriminate use of only broad-spectrum drugs. Although iAST recommended a greater number of drugs from the reserve group from the AWaRe classification in certain cases, particularly in empirical treatment, the software consistently demonstrated a higher rate of recommending drugs from the access group across all scenarios. Conversely, the recommendation rate for the watch group was consistently lower. In the current assessment, the ranking displayed all antibiotics routinely tested in the institution by kind of infection/microorganism. However, the software offers customization capabilities, enabling the selective display or concealment of specific antibiotics. This flexibility is determined by various factors, including the AWaRe classification, infection type, microorganism, patient treatment location, and patient age, which allows the unnecessary recommendation of broad-spectrum antibiotics when not required.

Recently, the INSPIRE randomized trial demonstrated how a computerized provider order entry bundle providing real-time recommendations for standard-spectrum antibiotics for patients with low multidrug-resistant organism risk coupled with feedback and education significantly reduced empiric extended-spectrum antibiotic use (28). Combining this kind of strategies with AI-based solutions such as the one here evaluated could improve the effectiveness of antimicrobial stewardship programs.

It is important to acknowledge that the present study presents limitations such as its non-prospective randomized design. Nonetheless, owing to the intrinsic features of the software and the antibiotic therapy workflow, the retrospective design facilitated the validation process, ensuring accuracy concerning the AST results. On the other hand, all infections included were monomicrobial and patients not under combination therapy were included in the study, which could represent a selection bias. Also, it is noteworthy that this software, similar to other previously developed algorithms, utilizes data from patients with culture-positive infections. However, there are instances of culture-negative infections, which result in limited data for refining the model. Nonetheless, the software offers supplementary value by assisting in the definitive treatment of such infections, even when microbiological documentation is unavailable. Additionally, the current study compares the software recommendations to the current practice in a single institution. Future studies could compare the software to the recommended antibiotic based on current clinical guidelines.

The standard practice of utilizing antibiograms to guide antibiotic selection in the global treatment of infections is well established worldwide. In this context, iAST emerges as a valuable tool capable of predicting AST results at least 48 h in advance of their issuance. It is also worth noting that iAST predictions are based on data from local epidemiology and scientific literature; however, for the choice of optimal antibiotic therapy, other patient variables such as risk factors, previous antimicrobial exposure, comorbidities, severity of infection, allergies, or drug interactions must be assessed by prescriber physicians.

In the current study, iAST showed outstanding performance in predicting antibiotic susceptibility compared to existing clinical practice. Thus, its integration as a tool for antibiotic prescription holds the promise to promote antibiotic stewardship. To enhance the impact of our findings, future research should consider prospectively investigating the incorporation of this system into infection management workflows and evaluating its impact on patient outcomes. Additionally, examining the potential integration of this system with other software platforms, such as electronic medical records and laboratory information systems, would provide valuable insights into optimizing its application in clinical settings. Other future considerations for evaluating the use of iAST include examining the interaction between AI and clinicians, exploring the potential for dependence on the system, and investigating its impact on standardized prescribing practices.

## SUPPLEMENTAL MATERIAL

Supplemental material aac.00777-24-s0001.pdf Figures S1 and S2; Tables S1 to S5.

ASM does not own the copyrights to Supplemental Material that may be linked to, or accessed through, an article. The authors have granted ASM a non-exclusive, world-wide license to publish the Supplemental Material files. Please contact the corresponding author directly for reuse.
