# Supplementary material for: Retrospective validation study of a machine learning-based software for empirical and organism-targeted antibiotic therapy selection
Source: Antimicrob Agents Chemother. 2024 Aug 28;68(10):e00777-24. doi: 10.1128/aac.00777-24 (PMC11460031; doi:10.1128/aac.00777-24)
Supplement: Supplemental material — Figures S1 and S2; Tables S1 to S5. [file aac.00777-24-s0001.pdf]

SUPPLEMENTARY MATERIAL:

**Content**	**Page**
**Supp. Figure 1:** iAST® interface screenshot	2
**Supp. Figure 2:** Microorganism heatmap	3
**Supp. Table S1:** Cumulative antibiograms from the most frequent Gram negative bacteria recovered from patients admitted to HM Hospitales from January to December 2022	4
**Supp. Table S2:** Cumulative antibiograms from the most frequent Gram positive bacteria recovered from patients admitted to HM Hospitales from January to December 2022	4
**Supp. Table S3:** Percentage of errors made in empirical treatment by the physician and the top three options in the iAST® ranking, by type of microorganism	5
**Supp. Table S4:** Percentage of errors made in organism-targeted treatment by the physician and the top three options in the iAST® ranking, by type of microorganism	6
**Supp. Table S5:** Frequency of antibiotics from the AWaRe reserve category prescribed by the doctor or recommended by the iAST® software, by type of molecule and infection.	7

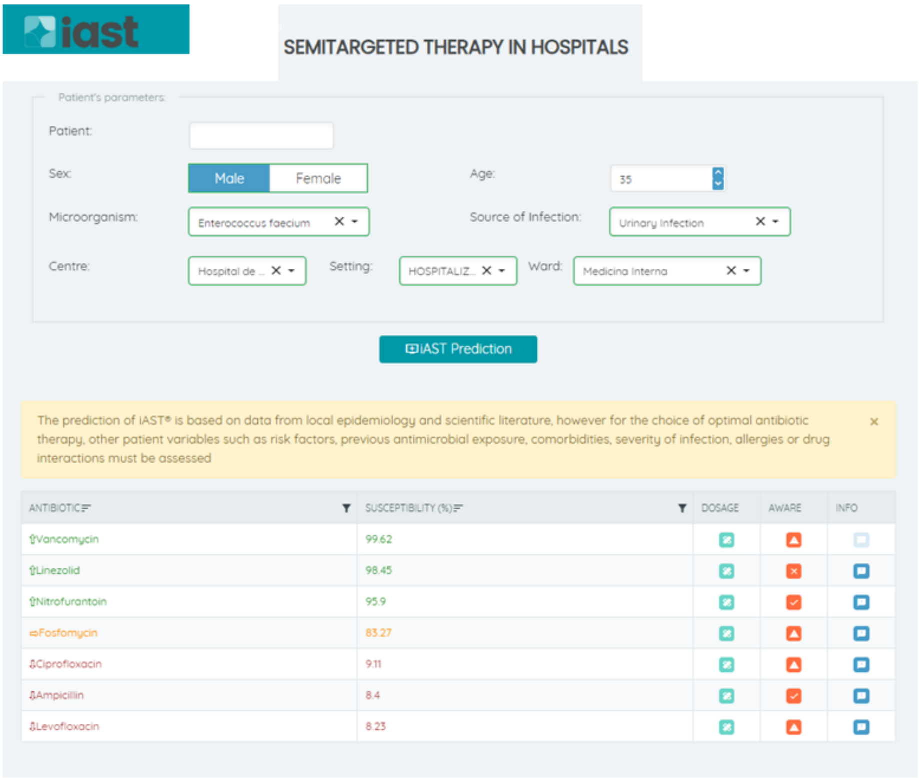

Supp. Figure 1: iAST® interface screenshot. The image displays essential software input parameters, including the patient's age and sex, among others. Additionally, it presents the output in the form of a computer-generated ranking of antibiotics based on the probability of microorganism coverage.

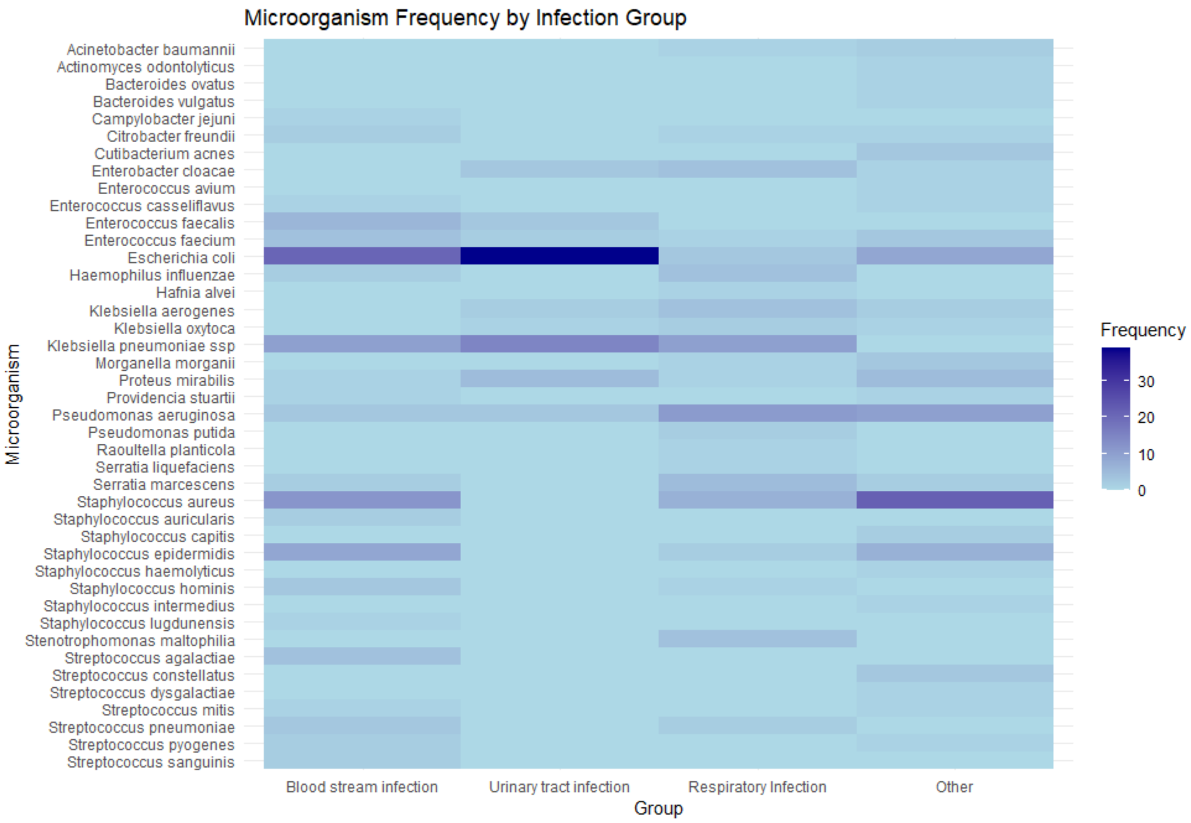

Supp. Figure 2: Microorganism heatmap. Distribution of microorganisms isolated from patients with infections in the study.

Supp. Table S1: Cumulative antibiograms from the most frequent Gram negative bacteria recovered from patients admitted to HM Hospitales from January to December 2022

Microorganism	N	AMP	AUG	TZP	C/T	CTX	CAZ	ERT	MER	CIP	LEV	SXT	FOS	NIF	COL	GM	TOB	AMK
Escherichia coli	12663	48.2%	80.6%	90.3%	NT	93.5%	NT	99.8%	99.9%	71.4%	NT	74.8%	97.5%	99.4%	97.3%	93.5%	89.0%	98.0%
Pseudomonas aeruginosa	1020	NT	NT	80.0%	93.1%	NT	83.7%	NT	83.9%	79.1%	NT	NT	NT	NT	80.4%	85.4%	95.2%	93.8%
Proteus mirabilis	973	67.9%	89.0%	97.7%	NT	98.3%	NT	96.5%	97.0%	77.4%	NT	61.8%	NT	NT	NT	90.5%	89.0%	95.5%
Klebsiella oxytoca	462	0.0%	81.3%	85.3%	NT	91.3%	NT	97.0%	100.0%	90.7%	NT	89.1%	NT	97.8%	95.6%	94.7%	84.0%	98.7%
Enterobacter cloacae	350	0.0%	0.3%	78.4%	NT	73.6%	NT	92.1%	98.6%	93.5%	NT	95.8%	NT	86.7%	92.0%	97.3%	95.5%	99.3%
Klebsiella pneumoniae	288	0.3%	80.5%	47.4%	NT	66,7%	NT	83.3%	83.3%	89.9%	NT	72.0%	NT	94.9%	100.0%	96.9%	83.3%	95.0%
Morganella morganii	264	0.8%	0.8%	97.9%	NT	68.3%	NT	98.7%	100.0%	78.1%	NT	77.2%	NT	NT	NT	88.8%	90.7%	95.7%
Citrobacter koseri	260	5.9%	92.9%	100.0%	NT	98.3%	NT	100.0%	100.0%	95.7%	NT	99.6%	NT	99.1%	90.3%	99.2%	99.3%	100.0%
Klebsiella aerogenes	249	0.0%	0.8%	68.6%	NT	68.3%	NT	91.5%	96.2%	88.8%	NT	93.5%	NT	86.0%	94.3%	95.9%	88.9%	97.2%
Haemophilus influenzae	237	80.0%	95.8%	NT	NT	99.5%	NT	NT	NT	96.0%	NT	NT	NT	NT	NT	100.0%	100.0%	NT
Stenotrophomonas maltophilia	235	NT	NT	NT	NT	NT	NT	NT	NT	NT	89.3%	98.2%	NT	NT	62.2%	12.0%	4.9%	2.9%
Campylobacter jejuni	221	NT	79.1%	NT	NT	NT	NT	NT	NT	11.4%	NT	NT	NT	NT	NT	NT	NT	NT
Serratia marcescens	204	0.0%	0.5%	96.9%	NT	82.1%	NT	99.4%	98.7%	94.6%	NT	98.4%	NT	NT	NT	96.5%	45.2%	77.5%

AMP,ampicillin; AUG, amoxicillin/clavulanic acid; TCP, piperacillin/tazobactam; C/T, ceftolozane/tazobactam; CTX, cefotaxime; CAZ, ceftaxidime; ERT, ertapenem; MER, meropenem; CIP, ciprofloxacin; LEV, levofloxacin; SXT, trimethoprim-sulfamethoxazole; FOS, fosfomycin; NIF, nitrofurantoin; COL, colistin; GM, gentamicin; TOB, tobramycin; AK, amikacin; NT, not tested.

Supp. Table S2: Cumulative antibiograms from the most frequent Gram positive bacteria recovered from patients admitted to HM Hospitales from January to December 2022

Microorganism	N	AMP	PEN	OXA	CIP	SXT	FOS	NIF	ERY	CD	VAN	LZD	DAP
Enterococcus faecalis	2938	99.2%	NT	NT	77.9%	NT	11.1%	4.6%	NT	NT	97,9%	98.4%	100.0%
Staphylococcus aureus	1624	NT	NT	83.5%	92.1%	97.1%	94.5%	87.7%	96.0%	97.5%	100.0%	99.8%	97.5%
Streptococcus agalactiae	1363	100.0%	100.0%	NT	88.9%	NT	17.6%	100.0%	94.0%	100.0%	100.0%	100.0%	100.0%
Staphylococcus epidermidis	649	NT	NT	25.0%	39.3%	61.9%	54.1%	26.1%	81.0%	98.8%	100.0%	91.1%	98.8%
Staphylococcus saprophyticus	498	NT	NT	100.0%	99.4%	95.5%	99.6%	90.2%	10.5%	98.0%	100.0%	96.0%	98.0%
Streptococcus pyogenes	450	100.0%	100.0%	NT	100.0%	NT	61.5%	100.0%	75.0%	100.0%	100.0%	100.0%	100.0%
Enterococcus faecium	232	16.1%	NT	NT	20.5%	NT	0.0%	0.0%	NT	NT	100.0%	98.2%	100.0%

AMP, ampicillin; PEN, penicillin, OXA, oxacillin; CIP, ciprofloxacin; SXT, trimethoprim-sulfamethoxazole; FOS, fosfomycin; NIF, nitrofurantoin; ERY, erythromycin; CD, clindamycin; VAN, vancomycin; LZD, linezolid; DAP, daptomycin; NT, not tested.

Supp. Table S3: Percentage of errors made in empirical treatment by the physician and the top three options in the iAST® ranking, by type of microorganism

Micoorganism	Doctor errors	iAST® 1st rank	iAST® 2nd rank	iAST® 3rd rank
Escherichia coli	25.4% (16/63)	6.3% (4/63)	6.3% (4/63)	6.3% (4/63)
Klebsiella pneumoniae	20% (7/35)	5.7% (2/35)	14.3% (5/35)	8.6% (3/35)
Staphylococcus aureus	15.8% (3/19)	0% (0/19)	0% (0/19)	0% (0/19)
Pseudomonas aeruginosa	52.9% (9/17)	5.9% (1/17)	29.4% (5/17)	5.9% (1/17)
Staphylococcus epidermidis	54.5% (6/11)	0% (0/11)	0% (0/11)	9.1% (1/11)
Enterococcus faecalis	33.3% (3/9)	0% (0/9)	0% (0/9)	0% (0/9)
Enterobacter cloacae	28.6% (2/7)	0% 0/9)	0% 0/9)	28.6% (2/9)
Enterococcus faecium	85.7% (6/7)	14.3% (1/7)	14.3% (1/7)	57.1% (4/7)
Proteus mirabilis	28.6% (2/7)	71.4% (5/7)	28.6% (2/7)	14.3% (1/7)
Serratia marcescens	42.9% (3/7)	0% (0/7)	0% (0/7)	0% (0/7)
Haemophilus influenzae	16.7% (1/6)	0% (0/6)	0% (0/6)	16.7% (1/6)
Klebsiella aerogenes	16.7% (1/6)	16.7% (1/6)	16.7% (1/6)	0% (0/6)
Streptococcus pneumoniae	20% (1/5)	20% (1/5)	0% (0/5)	0% (0/5)
Staphylococcus hominis	75% (3/4)	0% (0/4)	0% (0/4)	0% (0/4)
Stenotrophomonas maltophilia	100% (4/4)	75% (3/4)	75% (3/4)	50% (2/4)
Streptococcus agalactiae	0% (0/4)	0% (0/4)	0% (0/4)	0% (0/4)
Citrobacter freundii	33.3% (1/3)	0% (0/3)	0% (0/3)	33.3% (1/3)
Klebsiella oxytoca	66.7% (2/3)	0% (0/3)	0% (0/3)	0% (0/3)
Pseudomonas putida	0% (0/2)	0% (0/2)	0% (0/2)	0% (0/2)
Staphylococcus auricularis	0% (0/2)	0% (0/2)	0% (0/2)	0% (0/2)

Supp. Table S4: Percentage of errors made in organism-targeted treatment by the physician and the top three options in the iAST® ranking, by type of microorganism

Micoorganism	Doctor errors	iAST® 1st rank	iAST® 2nd rank	iAST® 3rd rank
Escherichia coli	13.9% (10/72)	2.8% (2/72)	5.6% (4/72)	8.3% (6/72)
Staphylococcus aureus	14.6% (6/41)	0% (0/41)	0% (0/41)	0% (0/41)
Klebsiella pneumoniae	17.1% (6/35)	2.9% (1/35)	8.6% (3/35)	5.7% (2/35)
Pseudomonas aeruginosa	11.1% (3/27)	0% (0/27)	3.7% (1/27)	3.7% (1/27)
Staphylococcus epidermidis	22.2% (4/18)	0% (0/18)	0% (0/18)	5.6% (1/18)
Proteus mirabilis	8.3% (1/12)	8.3% (1/12)	33.3% (4/12)	16.7% (2/12)
Enterococcus faecium	30% (3/10)	0% (0/10)	0% (0/10)	40% (4/10)
Enterococcus faecalis	22.2% (2/9)	0% (0/9)	0% (0/9)	0% (0/9)
Serratia marcescens	11.1% (1/9)	0% (0/9)	0% (0/9)	0% (0/9)
Enterobacter cloacae	50% (4/8)	0% (0/8)	0% (0/8)	25% (2/8)
Klebsiella aerogenes	0% (0/8)	0% (0/8)	12.5% (1/8)	0% (0/8)
Haemophilus influenzae	16.7% (1/6)	16.7% (1/6)	0% (0/6)	16.7% (1/6)
Streptococcus pneumoniae	0% (0/5)	0% (0/5)	0% (0/5)	0% (0/5)
Citrobacter freundii	25% (1/4)	0% (0/4)	0% (0/4)	0% (0/4)
Klebsiella oxytoca	50% (2/4)	0% (0/4)	0% (0/4)	0% (0/4)
Morganella morganii	25% (1/4)	0% (0/4)	0% (0/4)	0% (0/4)
Staphylococcus hominis	25% (1/4)	0% (0/4)	0% (0/4)	0% (0/4)
Stenotrophomonas maltophilia	75% (3/4)	25% (1/4)	75% (3/4)	75% (3/4)
Streptococcus agalactiae	0% (0/4)	0% (0/4)	0% (0/4)	0% (0/4)
Acinetobacter baumannii	33.3% (1/3)	33.3% (1/3)	33.3% (1/3)	66.7% (2/3)

Supp. Table S5: Frequency of antibiotics from the AWaRe reserve category prescribed by the doctor or recommended by the iAST® software, by type of molecule and infecti

	Empirical therapy				Organism-targeted therapy			
	**Doctor**	**iAST® 1st rank**	**iAST® 2nd rank**	**iAST® 3rd rank**	**Doctor**	**iAST® 1st rank**	**iAST® 2nd rank**	**iAST® 3rd ran**
**Antibiotics**								
AZT	5.9% (1/17)	-	-	-	1.5% (1/66)	-	-	-
CAZ/AVI	5.9% (1/17)	-	-	-	4.5% (3/66)	-	-	-
C/T	-	-	-	-	1.5% (1/66)	24.4% 19/78)	3.2% (2/62)	-
COL	-	-	-	-	1.5% (1/66)	2.6% (2/78)	3.2% (2/62)	2,9% (2/70)
CPT	-	-	-	-	1.5% (1/66)	-	-	-
DAP	64.7% (11/17)	44.8% (13/29)	64.4% (29/45)	59.6% (31/52)	37.9% (25/66)	8.9% (7/78)	40.3% (25/62)	58.6% (41/70)
LZD	23,5% (4/17)	55.2% (16/29)	35.6% (16/45)	40.4% (21/52)	50% (33/66)	41.1% (50/78)	53.2% (33/62)	38.6% (27/70)
TIG	-	-	-	-	1.5% (1/66)	-	-	-
**Type of infection**								
BSI	70.6% (12/17)	51.7% (15/29)	84.4% (38/45)	79.9% (40/52)	37.9% (25/66)	29.5% (23/78)	48.4% (30/62)	41.4% (29/70)
Pneumonia/VAT	17.6% (3/17)	48.3% (14/29)	15.6% (7/45)	20.1% (12/52)	16.7% (11/66)	15.4% (12/78)	19.4% (12/62)	15.7% (15/70)
UTI	11.8% (2/17)	-	-	-	6.1% (4/66)	1.3% (1/78)	3.2% (2/62)	1.4% (1/70)
Other	-	-	-	-	39.4% (26/66)	53.8% (42/78)	29.0% (18/62)	41.4% (29/70)

AZT, aztreonam; CAZ/AVI, ceftazidime/avibactam; C/T, ceftolozane/tazobactam; COL, colistin; CPT, ceftaroline; DAP, daptomycin; LZD, linezolid; TIG, tigecycline; BSI, blood stream infection; VAT, ventilator associated tracheobronchitis; UTI, urinary tract infection.
